# Significance of early diagnosis and surgical management in treating *Mycobacterium immunogenum*-related pyogenic extensor tenosynovitis: a case report

**DOI:** 10.1186/s12879-024-09249-5

**Published:** 2024-04-12

**Authors:** Tomohide Okinaka, Kenjiro Fujimura, Yuka Hamasaki, Yuichi Hasegawa, Takashi Matono

**Affiliations:** 1 Department of Infectious Diseases, Aso Iizuka Hospital, Iizuka, Fukuoka, Japan; 2 Department of Orthopedic Surgery, Aso Iizuka Hospital, Fukuoka, Japan; 3 Department of Dermatology, Inatsuki Hospital, Fukuoka, Japan

**Keywords:** *Mycobacterium immunogenum*, Pyogenic extensor tenosynovitis, Nontuberculous mycobacterium, Rapidly growing mycobacteria

## Abstract

**Background:**

Non-tuberculous mycobacteria (NTM) are environmental organisms that are increasingly contributing to human infections. *Mycobacterium immunogenum*, a variant of NTM discovered in 2001, is a rapidly growing mycobacterium that exhibits multidrug resistance. Reports of infections caused by this organism, particularly tenosynovitis in the musculoskeletal system, are limited.

**Case presentation:**

A 71-year-old female with vesicular pemphigus, undergoing immunosuppressive therapy, presented with a progressively enlarging tumour on the dorsum of her right hand, along with erythematous papules that extended across her right forearm. The specimens of skin tissues and blood cultures revealed the presence of *M. immunogenum*. Magnetic resonance imaging evaluation led to the diagnosis of pyogenic extensor tenosynovitis. A multidrug regimen, comprising amikacin and clarithromycin, was initiated, followed by synovectomy. The patient underwent a course of 180 days of antimicrobial therapy and demonstrated no signs of disease recurrence one year after treatment completion.

**Conclusion:**

Early diagnosis and surgical intervention are crucial to prevent the adverse prognostic implications of pyogenic extensor tenosynovitis caused by *M. immunogenum*. Effective management requires precise microbial identification and susceptibility testing, necessitating collaborative engagement with microbiological laboratories.

## Background

Non-tuberculous mycobacteria (NTM) are ubiquitously distributed in the environment and exert various effects on human and animal health. *Mycobacterium immunogenum* was first identified as a rapidly growing mycobacteria by Wilson et al. in 2001 [1]. This bacterium belongs to the *Mycobacterium abscessus*-*Mycobacterium chelonae* group. It exhibits only an 8 bp difference in the 16 S ribosomal DNA sequence from that of *M. abscessus* and a 10 bp difference from that of *M. chelonae* [2]. It shares similar biological characteristics and antibiotic susceptibilities with *M. abscessus* and *M. chelonae*. However, *M. abscessus* is clarithromycin (CAM)-resistant due to erythromycin ribosomal resistance methylase (*erm*) gene expression, while *M. chelonae* is CAM-sensitive due to the absence of the *erm* gene. Thus, accurate species identification and susceptibility testing are crucial for selecting effective treatment regimen. *M. immunogenum* can inhabit various environments including soil, dust, water, aerosols, metalworking fluids, and surgical instruments. Although it is associated with a range of diseases such as skin and soft tissue infections, keratitis, and hypersensitivity pneumonitis [3, 4], reports on musculoskeletal infections such as tenosynovitis are extremely rare. According to previous studies [5], extrapulmonary NTM infections account for approximately 10% of all NTM infections, the incidence of NTM-induced tenosynovitis is even lower, and specific epidemiological data are insufficient. Most cases with tenosynovitis are caused by *M. marinum*, *M*. *avium*-*intracellulare* (MAI) complex, and *M. chelonae* [6, 7], whereas the clinical and treatment courses of tenosynovitis caused by *M. immunogenum* have not been well-described. We report a patient with pyogenic extensor tenosynovitis of the right finger who was receiving immunosuppressive therapy for vesicular pemphigus.

## Case presentation

**Fig. 1:**
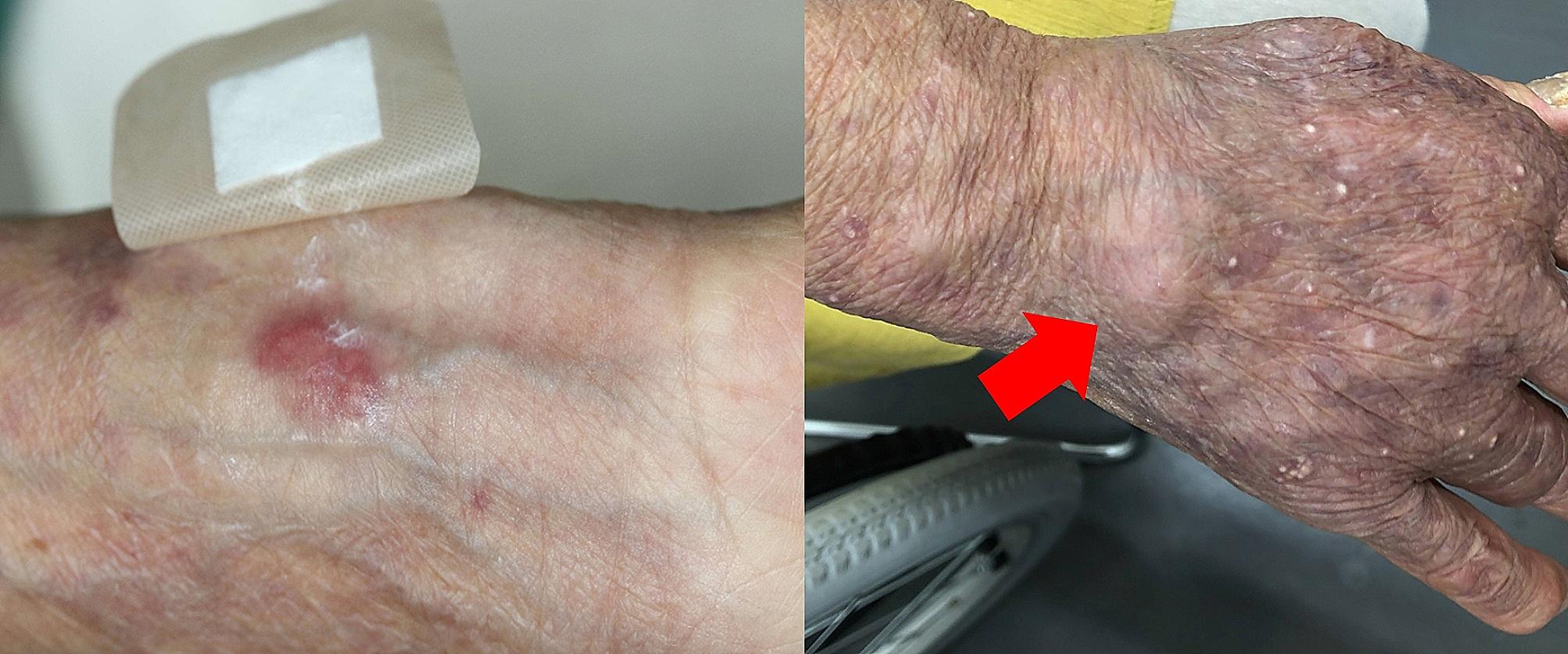
Skin lesions on right forearm and hand Erythematous papules on the medial side of the right forearm and painless mass on the dorsal surface of the right hand (red arrow). The scattered white bullous lesions on the dorsum of the right hand are bullous pemphigoids

**Fig. 2:**
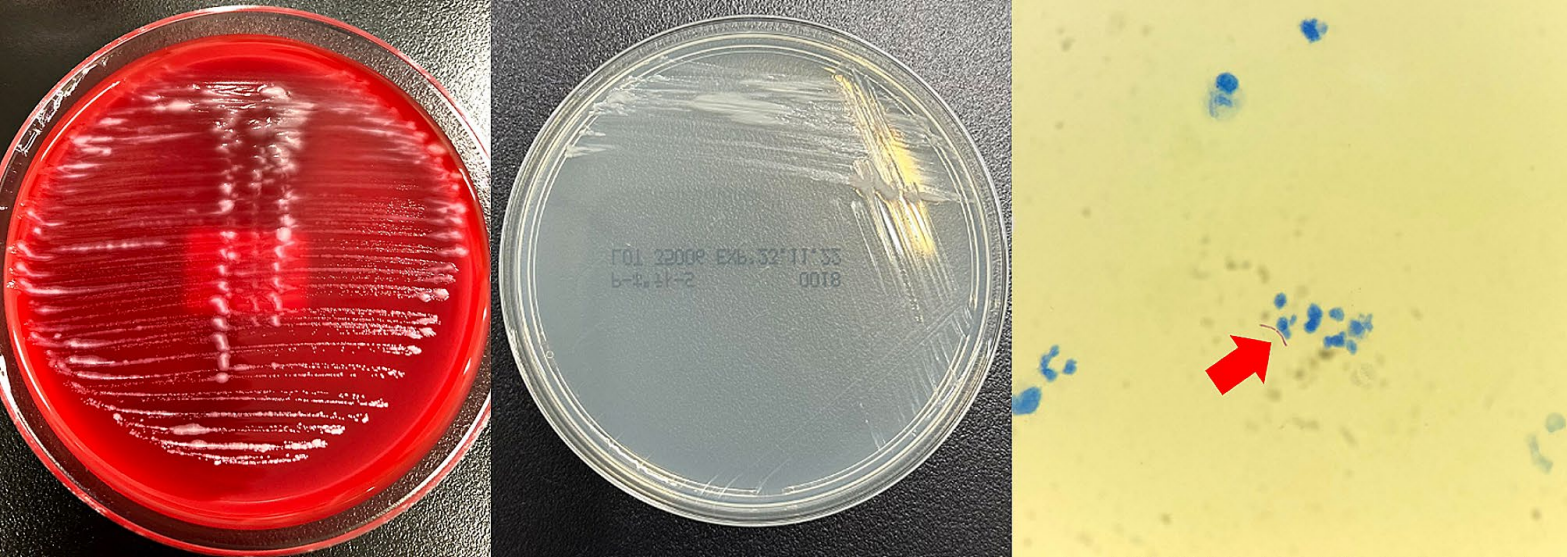
Colonies and Ziehl-Neelsen staining of *Mycobacterium immunogenum* Colonies of *Mycobacterium immunogenum* grown on blood agar medium and on potato dextrose agar medium. Ziehl-Neelsen staining of a specimen taken from the right-hand tumour site reveals the presence of *Mycobacterium immunogenum*, which is indicated with a red arrow

**Fig. 3:**
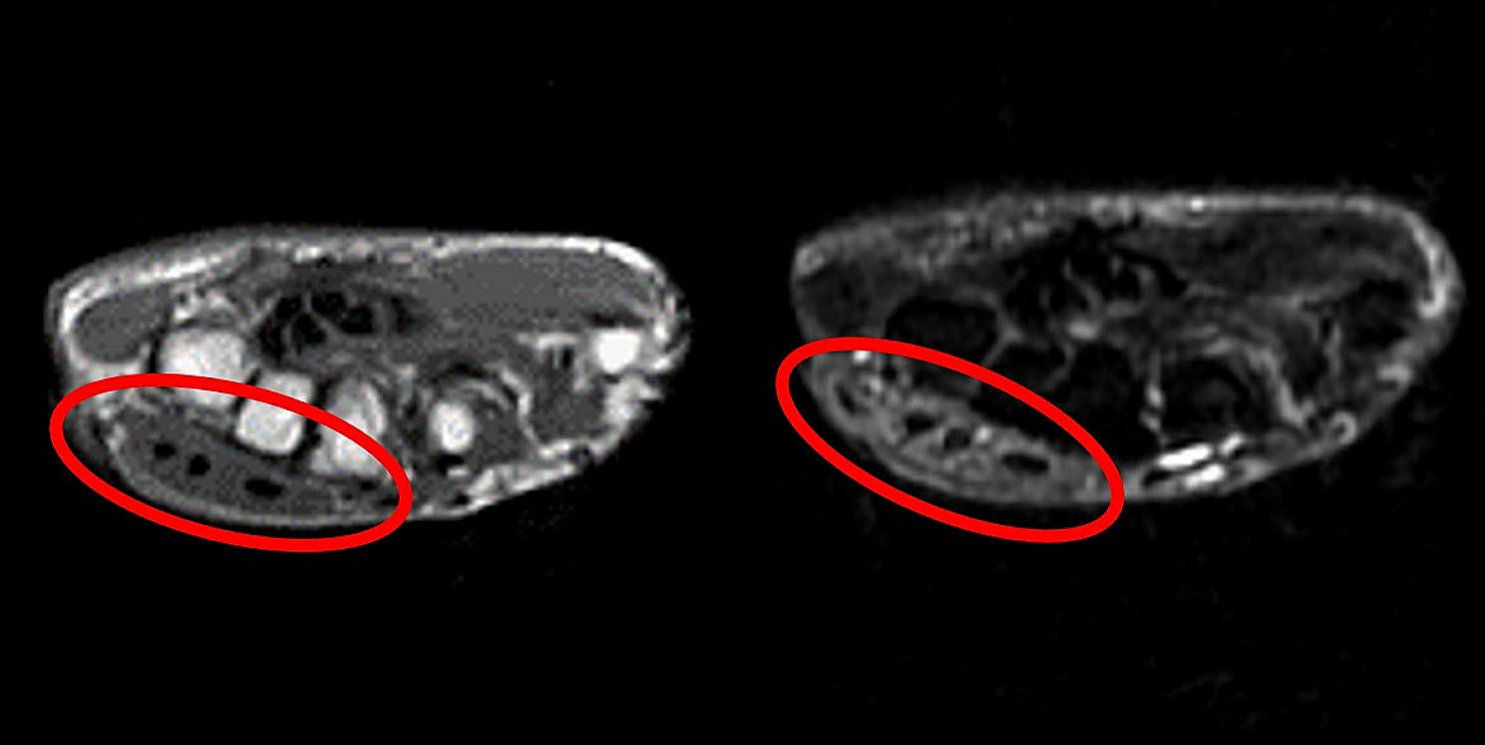
Magnetic resonance imaging of the right hand (left: T1-weighted images, right: T2-weighted images) Magnetic resonance imaging shows pyogenic extensor tenosynovitis around the extensor tendons of the right hand, which is marked by a red circle

**Fig. 4:**
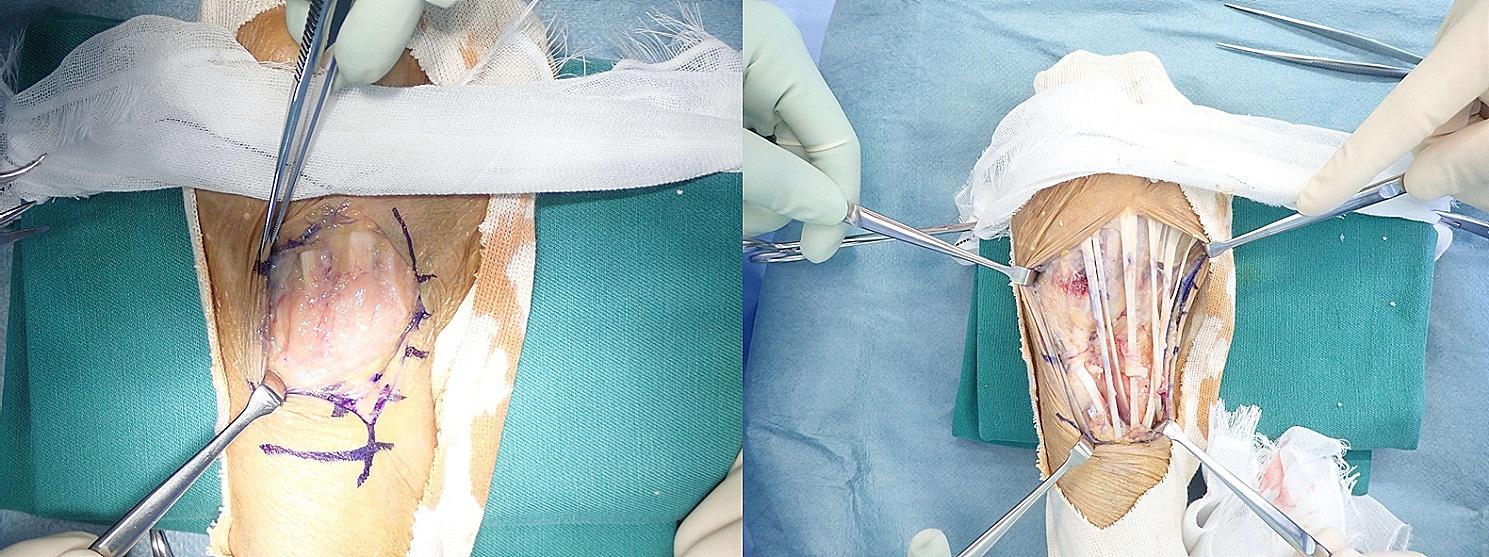
Findings before and after synovectomy White cake-like tissue is present within the synovium, and synovitis is present proximal to the extensor digitorum flavum. A Z-shaped incision is made through the extensor digitorum flavum and a synovectomy is performed to ensure that no remnants

A 71-year-old female with vesicular pemphigus presented with a 2.5-months history of gradually increasing/enlarging erythematous papules on her right forearm and a tumour on the dorsum of her right hand (Fig. 1). She was on oral betamethasone (1.65 mg/day), mizoribine (100 mg/day), and methotrexate (3 mg/week) for the treatment of vesicular pemphigus. The patient had a history of occasional weeding without gloves but no involvement in gardening, soil manipulation, or handling fish tanks. Laboratory tests showed a white blood cell count of 10,070/µL and a C-reactive protein level of 4.4 mg/L. Chest computed tomography showed no signs of pulmonary inflammation. Ziehl-Neelsen staining of the specimen from tumour biopsy yielded positive results. Colonies grew in blood agar and potato dextrose agar media from the biopsy specimens of papules and tumour (Fig. 2). The microbial culture conditions were set at a temperature of 37 °C for the blood agar medium, while the potato dextrose agar medium was maintained at 27 °C. The colonies under consideration were observed to develop within a span of seven days for both media and were identified as *M. immunogenum* using matrix-assisted laser desorption/ionization-time of flight mass spectrometry (MALDI-TOF-MS) (MALDI Biotyper® by Bruker Daltonics). The results of 16 S rRNA gene analysis, rpoB gene analysis, and nucleic acid chromatography, which were conducted by BML Inc, confirmed that the isolates were *M. immunogenum*. No organisms were detected in the cultures of blood specimens tested using BACTEC Myco/F bottles (Becton Dickinson, Japan). Treatment with amikacin (AMK) and CAM was initiated based on susceptibility testing following the guidelines of the Clinical and Laboratory Standards Institute M24, 3rd ed. 2018, conducted by Special Reference Laboratories, Inc. (Table 1). Magnetic resonance imaging (MRI) revealed low signal intensity on T1-weighted images and high signal intensity on T2-weighted images around the extensor tendons of the second to fifth metacarpal bone level, leading to a diagnosis of pyogenic extensor tenosynovitis of the right hand, without rice body findings (Fig. 3). On the 18th day of hospitalization, synovectomy was performed, and the infected lesion was excised as much as possible (Fig. 4). After the patient was transferred to a convalescent hospital on day 46, the dosing regimen of AMK was changed to thrice weekly starting from day 84. Blood concentrations of AMK were monitored, and no adverse events occurred under the combination therapy. Home treatment was continued through visiting nursing care after discharge on day 118. The combination therapy was stopped on day 180, and no recurrence has been observed for more than a year.

**Table 1:** Susceptibility testing for *Mycobacterium immunogenum*

Antibiotics	MIC(µg/mL)	MIC break point from CLSI M24(µg/mL)
Amikacin	8	16
Tobramycin	8	2
Imipenem	16	4
Moxifloxacin	8	1
Trimethoprim Sulfamethoxazol	> 152/8	2/38
Doxycycline	> 16	1
Meropenem	> 64	4
Linezolid	32	8
Clarithromycin	0.5	2

## Discussion and conclusions

We describe a case of pyogenic extensor tenosynovitis of the right finger caused by *M. immunogenum*. To our knowledge, the only pre-existing report on tenosynovitis caused by *M. immunogenum* was a case of flexor tenosynovitis reported by Aryee et al. [8]. Here, we discuss the diagnostic characteristics of NTM-related tenosynovitis, detection of *M. immunogenum*, and treatment of *M. immunogenum*-associated infections.

First, tenosynovitis caused by NTM is often seen in the hands and wrists, likely owing to the higher probability of penetrating injuries [9–12]. Furthermore, cellular immunity, mainly phagocytosis and Th1 cells, plays an important role in the defence against infection by NTM, an intracellular parasite. The risk of infection is particularly high in immunocompromised patients. Although our patient had a habit of weeding without gloves, there was no event of obvious injury. Thus, this case suggests that even if there is no clear history of injury, NTM should be considered as a causative microbe in patients with predisposing factors of cellular immune deficiency. As observed in the patient in this case, the chronic clinical course and the swelling in the affected hand without systemic symptoms such as fever, align with the previously reported cases of NTM tenosynovitis [13]. The diagnosis of NTM tenosynovitis is often delayed. For example, Napaumpaiporn et al. reported that the average duration from symptom onset to diagnosis was 16 weeks [14]. However, delay in diagnosis and treatment can worsen functional outcomes. According to a study by David et al., the prognosis worsens if the diagnosis of NTM is delayed by more than 4 months [15]. Thus, early diagnosis is essential for a good prognosis. In our case, the diagnosis was achieved relatively earlier (approximately 10 weeks after the symptom onset) than that in the previous report [14]. This may have contributed to the successful treatment. It is vital to consider NTM-related skin and soft tissue or musculoskeletal infections in individuals with risk factors, including cosmetic surgery, piercings, tattoos, mesotherapy, acupuncture, intra-articular steroid injections, intravascular devices (such as central venous catheters), peritoneal dialysis catheters, injuries from fresh or saltwater, and immunodeficiency [16, 17]. Additionally, although not observed in the present case, the presence of rice bodies on the MRI may assist in the diagnosis of NTM tenosynovitis [7].

Second, clinicians should know the appropriate culture conditions for acid-fast bacterial growth to detect NTM effectively. The rapidly growing mycobacteria (RGM) group, including *M. immunogenum*, reaches mature colonies within a week of culture. While the majority of slowly growing mycobacteria thrive best at 35–37 °C, RGMs prefer a relatively lower optimal temperature of 28–30 °C. Hence, cultures under lower temperature conditions should be additionally conducted when an extrapulmonary NTM infection is clinically suspected. Because the RGM-related infections generally require long-term treatment and species-specific combination therapy [18], species identification and drug-susceptibility testing are crucial. However, as the taxonomy of NTM is diverse, accurate and prompt species-level detection is often challenging in the clinical settings. Commonly used commercial rapid detection methods using PCR analysis in the clinical laboratory occasionally misdiagnose the rare NTM as MAI complex [19]. Therefore, sequencing of housekeeping genes, such as 16 S rRNA, rpoB, and hsp65, is necessary for accurate microbial diagnosis [20]. Recently, the value and accuracy of MALDI in NTM diagnosis has increased. In our case, identification was achieved using MALDI-TOF-MS (MALDI Biotyper®, Bruker Daltonics) and was further confirmed by 16 S rRNA and rpoB gene analyses and nucleic acid chromatography.

Third, based on the recommendations for treating severe NTM skin and soft tissue infections with at least two drugs [21], this case was treated with a regimen of CAM and AMK. *M. immunogenum*, belongs to the *M. chelonae* complex group, which typically does not exhibit resistance to CAM because of its non-functional *erm* gene [22]. We performed two minimum inhibitory concentration (MIC) tests for CAM on days 3 and 14 to confirm their susceptibility. Although there are no specific guidelines for the intermittent dosing of AMK for *M. immunogenum*, AMK at 25 mg/kg thrice weekly is a probable treatment regimen for *M. abscessus*, albeit with potential tolerability issues for extended use [18]. The treatment duration for *M. immunogenum* infection varies depending on the infected organ and patient’s immune conditions. While some cases of brain abscesses resolve with a 2-week treatment, soft tissue infections require up to 12 months [23]. There is no established treatment duration for NTM-induced upper limb tenosynovitis, but the median treatment duration ranges from 6 to 9.8 months [13, 24, 25]. Our patient was treated with antimicrobials for 6 months. There are reports advocating treatment solely with antibiotics to avoid complications from extensive tendon debridement [13], although more reports exist on combined surgical and antibiotic treatments. We believe that synovectomy, performed for infected tissue removal, can be useful for reducing treatment duration and side effects.

In conclusion, although tenosynovitis due to *M. immunogenum* is relatively rare, delayed diagnosis and treatment can severely affect a patient’s quality of life. For early and accurate diagnosis, collaboration of clinicians with microbiology technicians and optimum culture conditions are vital when the infection caused by NTM is suspected, particularly in immunocompromised hosts. Furthermore, clinicians should consider the benefits of aggressive surgical intervention for achieving good clinical outcome.

## Data Availability

The datasets used and analysed during the current study is available from the corresponding author upon reasonable request.

## Abbreviations

NTM: non-tuberculous mycobacteria
MAI: *M. avium-intracellulare*
RGM: rapidly growing mycobacteria
*erm*: erythromycin ribosomal resistance methylase
CAM: clarithromycin
AMK: amikacin
MIC: minimum inhibitory concentration
MALDI-TOF-MS: matrix-assisted laser desorption/ionization-time of flight mass spectrometry
MRI: Magnetic Resonance Imaging
